# Concomitant Presence of Ovarian Tumors (Teratoma and Granulosa Cell Tumor), and Pyometra in an English Bulldog Female Dog: A Case Report

**DOI:** 10.3389/fvets.2019.00500

**Published:** 2020-01-14

**Authors:** Carlos A. Oviedo-Peñata, Luis Hincapie, Carlos Riaño-Benavides, Juan G. Maldonado-Estrada

**Affiliations:** ^1^Faculty of Veterinary Medicine and Zootechny, University of Cordoba, Monteria, Colombia; ^2^Surgery and Theriogenology Branch OHVRI-Group, School of Veterinary Medicine, University of Antioquia, Medellin, Colombia

**Keywords:** dog, ovary, neoplasia, tumor, uterus

## Abstract

**Background:** The diagnosis of ovarian tumors in dogs is usually complicated because the clinical signs can be very discrete and can be easily confused with other diseases. There are few reports of ovarian tumors with different cellular characteristics in the same dog. Our objective was to describe an unusual case of the concomitant presence of ovarian teratoma and granulosa cell tumors in a female dog presenting symptoms compatible with pyometra at clinical consultation.

**Clinical history:** A non-spayed 6-years-old female English Bulldog was attended at the consultation, with no history of previous steroid hormonal treatment. The dog had presented regular estrus every 6 months; 3 months elapsed between the last estrus and consultation. The dog had presented vulvar discharge for more than 2 weeks.

**Clinical and laboratory findings:** the patient presented a slightly pale oral mucosa, decay, vulvar edema, and mucous-purulent uterine discharge. The ultrasound examination revealed the presence of neoformations in the ovaries, and evidence of cystic endometrial hyperplasia-pyometra in the uterus.

**Treatment:** We performed a ventral ovariohysterectomy. During the surgical procedure, it was found several masses in the left and right ovaries, exhibiting characteristics of other tissues different from ovarian tissue. All samples were sent for histopathological examination. The diagnosis was a granulosa cell tumor in the left ovary and a well-differentiated teratoma in the right ovary.

**Conclusion:** Practitioners must improve the use of diagnostic tools when attending non-spayed dogs at advanced ages (more than 6 years old), which would probably be at high risk of suffering from undetected ovarian tumors, some of them with malignancy behavior.

## Background

Granulosa cell tumor (GCT) is reported as one of the most frequent ovarian neoplasia in female dogs (1). Teratomas are another type of tumor originating from pluripotent or totipotent cells remaining from the embryonic notochord. These tumors could present a solid or cystic macroscopic aspect and contain germinal cells that originate from the three germinative layers, (2). It can be found: hair, sebaceous glands, nervous tissue (ectodermal origin), cartilage, tooth, muscle (mesodermal origin), and intestinal or respiratory epithelium (endodermal origin) (3, 4) The ovarian teratoma is more frequently found in dogs compared to cats. It represents 9.7 and 2% of ovarian tumors (5) and primary ovarian tumors (6), respectively. In dogs, ovarian teratoma is mostly benign and well-differentiated (5, 7), although there exists a report of malignant teratoma (8). Due to its non-specific clinical signs, the clinical diagnosis of teratoma is challenging. Ovarian teratoma is frequently found, on average, in 6.5 year-old dogs (range: 20 months to 13 years) (7, 9).

Granulosa cell tumor (GCT) originates from granulosa cells of ovarian follicles (10) and can present solid or cystic follicular patterns (1, 10). GCT can exert hormonal activity resulting in estrogen (11), anti-Mullerian hormone (12), and alpha-inhibin secretion (13). The microscopic histopathological exam is mandatory for the definite diagnosis of GCT (10). The election treatment for GCT and teratoma is ovariohysterectomy (14). This report aims to describe a concomitant presence of ovarian teratoma, GCT, and pyometra in an English Bulldog female found intra-operatory in elective surgery of a dog suffering from pyometra. A comprehensive review of the available literature is also discussed.

## Case Presentation

### Clinical History

A 6-years-old, nulliparous, intact English Bulldog female was referred to the University of Antioquia Veterinary Teaching Hospital (Small animal clinic). The dog weighed 24.3 kg, had complete immunoprophylaxis and antiparasitic treatments, and was fed with commercial dog food. The owner reported no previous hormonal treatment, and 3 months had elapsed after its last standing estrus, that lasted 21 days. Two weeks before the consultation, the dog started with fetid vaginal discharge.

### Physical Examination

Rectal temperature, respiratory rate, heart rate, and capillary refill time were 38.3°C, 25 rpm (with increased vesicular murmur), 135 b.p.m., and 1 s, respectively, with a strong pulse, rhythmic, and concordant. The oral mucosa was found slightly pale, wet, and brilliant. The dog exhibited a docile attitude, had a 2.5 Body condition score (in a 1–5 scale), and was down although she was able to feed with commercial concentrate and drink water. An increased size of mammary glands, edematous vulva, and vulvar secretion of fetid sanguine-purulent aspect were observed. At palpation, the patient exhibited abdominal pain.

### Diagnostic Aids

The dog was referred to with the results of blood cell counts (The blood cell counts are presented in Table 1). A blood sample was taken by radial vein puncture for measurements of plasma proteins, activated partial thromboplastin-time, ALT, and creatinine, using an IDEXX Pro Cyte Dx^®^ automated equipment (DEXX Laboratories, Westbrook, MA, USA). The results of blood cell counts were indicative of normocytic normochromic anemia. Also, a vaginal cytology smear was prepared on microscopic slides and stained with Hematoxylin & Eosin, resulting in predominant degenerated polymorphonuclear neutrophils, and erythrocytes. All exams were performed in the Clinical Pathology Laboratory, School of Veterinary Medicine, University of Antioquia. No hormonal measurements were performed.

**Table 1:** Results of blood cell counts and blood biochemistry exams.

**Parameter**	**Units**	**Result**	**Reference value**
White blood cells	Cells × 10^3^/μl	11.02	6.0–17.0
Lymphocytes	Cells × 10^3^/μl	1.02	1.0–4.8
Intermediate cells	Cells × 10^3^/μl	1.5	0.2–1.5
Granulocytes	Cells × 10^3^/μl	8.27	3.0–12.0
Lymphocytes	%	12	12–30
Monocytes	%	0	2–4
Neutrophils	%	73	62–87
Eosinophils	%	2	0–10
Basophils	%	0	0–1
Bands	%	17	–
Erythrocytes	Cells × 10^3^/μl	4.73	5.5–8.5
Hemoglobin	g/dl	11.2	12.0–18.0
Hematocrit	%	35.65	37.0–55.0
Median corpuscular volume	fl	75	60.0–77.0
Median corpuscular hemoglobin	Pg	24.3	19.5–24.5
Median corpuscular hemoglobin concentration	g/dl	33.9	31.0–34.0
Red cells distribution wide	%	13.8	–
Platelet counts	Cells × 10^3^/μl	217	200–500
Platelet package	%	0.32	–
Mean platelet volume	fl	14.8	3.9–11.1
Platelet distribution wide	%	42.3	–
Plasma proteins	g/dl	7.2	6.0–7.5
Reticulocytes	%	9.460	>60.000
Activated partial thromboplastin time	Sec.	22	12–22
ALT/GPT	U/L	18.2	18–81
Creatinine	mg/dl	1.02	0.6–1.4
Serum aspect	Normal		
Peripheral blood smear	Normal red cells and platelets		

### Clinical Findings

**Figure 1:**
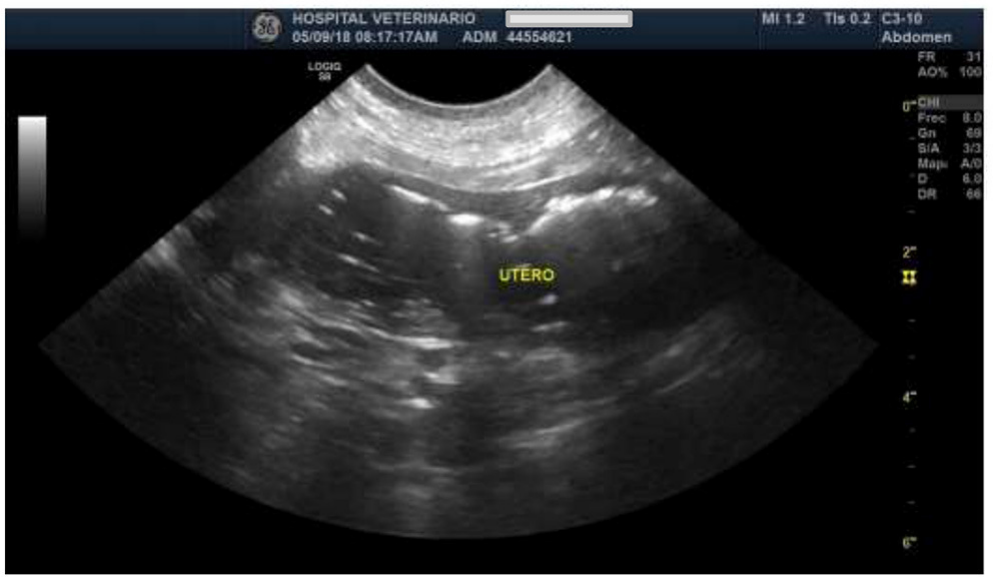
Ultrasound image of the uterus, indicating the increased size and tighter uterine wall of irregular contour.

At the abdominal ultrasound examination, both ovaries showed increased size with fine-heterogeneous hyperechoic structure, a 3.0 × 2.5 cm cyst not compromising the ovarian silhouette. The uterine wall was tight, exhibiting irregular contour. Also, homogeneous intramural cystic structures and a hypoechoic luminal fluid associated with cystic walls were found (Figure 1). A presumptive diagnosis of open pyometra was established. Ovariohysterectomy was prescribed, and the dog was prepared for surgery.

### Surgical Procedure

For preparation, the dog was subjected to 12 h of solid food and 8 h of liquids fasting. According to the protocol of University of Antioquia Veterinary Teaching Hospital: Pre-anesthesia was performed with Tramadol (Tramadol, MK, 2 mg/kg/SC), Acepromazine (Tranquilan, Zoo laboratories, 0.02 mg/kg/IM), Meloxicam (Meloxic, Provet, 0.2 mg/kg/EV), Dipyrone (Colivet, Provet 28 mg/kg/EV). Cefalotine (Cefalotine, Vitalis, 20 mg/kg/IV) was administered before initiating the surgery. Anesthesia induction was achieved with Propofol (Propofol 1% MCT/LCT, Fresenius Kabi, 2.6 mg/kg/IV). The deep anesthetic level was maintained with 2 CAM isoflurane. Also, a lumbosacral epidural blockade was done with epinephrine-free Lidocaine (Lidocaine, Virbac Laboratories, 1 mg/kg). The surgical plans were according to the conventional approach through line alba. All the surgical plans were performed with no complications.

### Macroscopic Findings

**Figure 2:**
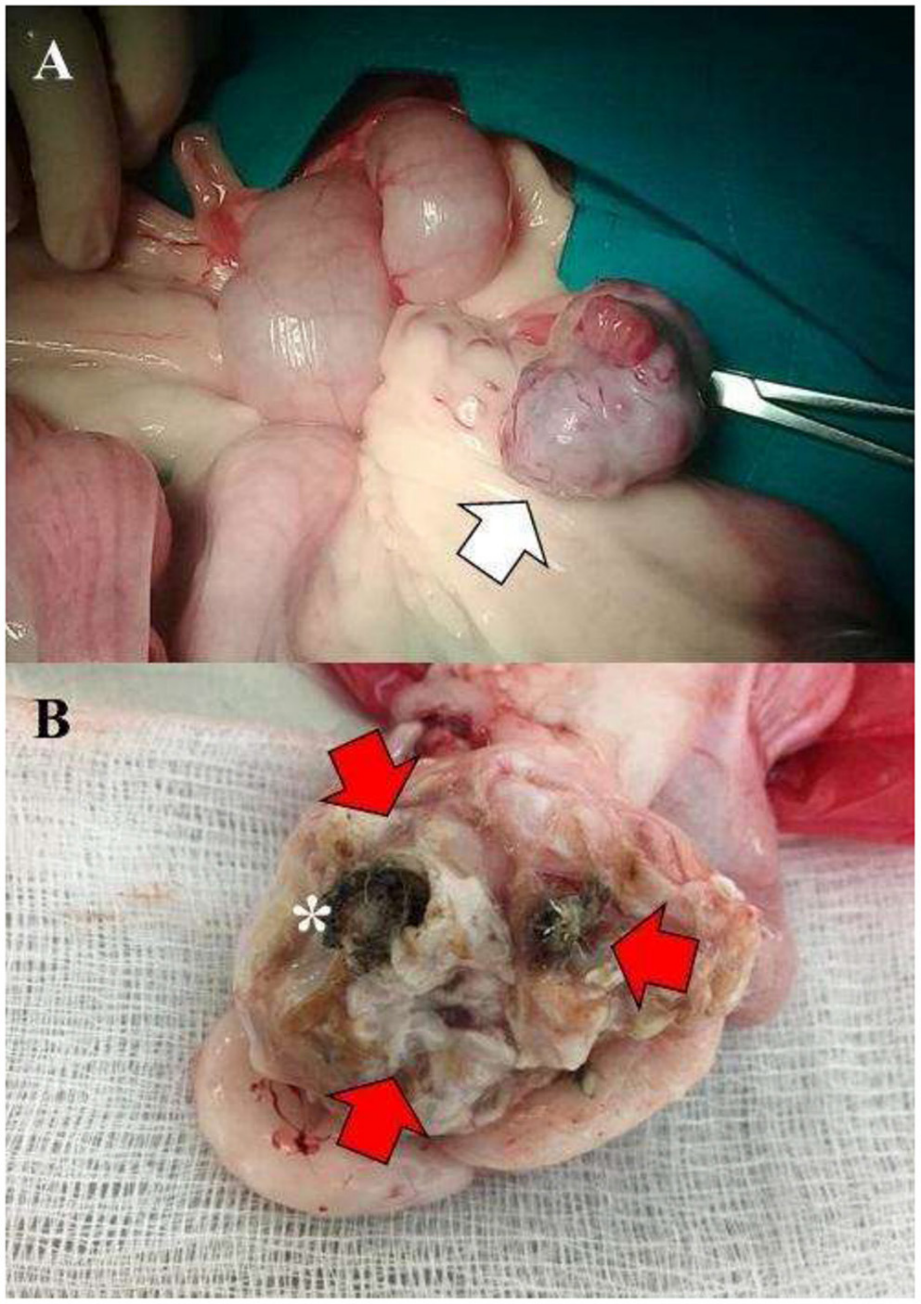
**(A)** Polycystic left ovary with evidently increased size (3.0 × 2.5 cm) (White arrow). **(B)** Right ovary exhibiting hair, cartilage (Red arrows), and bone tissue (asterisk).

The uterus presented a slightly symmetrical distention, occupied with purulent material. The ovaries were found polycystic, measuring 3.0 × 2.5 cm, with normal color and aspect (Figure 2A). In a sagittal cutting of the right and left ovaries, hair, and bone tissue (Figure 2B), and polycystic structures were found, respectively. Samples taken from the uterus and both ovaries were fixed in 10% formaldehyde and were sent to the Laboratory of Animal Pathology, the University of Antioquia, and processed for histopathological evaluation with hematoxylin-eosin staining. Diagnosis was performed by an experienced pathologist with more than 20 years in microscopic histopathologic diagnoses.

### Post-operative Follow-Up

Post-operative treatment included: Ampicillin, Metronidazole, Ranitidine, Tramadol, and Meloxicam at the recommended doses and treatment schedule. Also, 0.5% of topical chlorhexidine was prescribed for the healing of the surgical incision. The sutures were removed on the 20th post-operative day. The dog recovered with no complications up to the 20th post-operative day.

### Microscopic Findings

**Figure 3:**
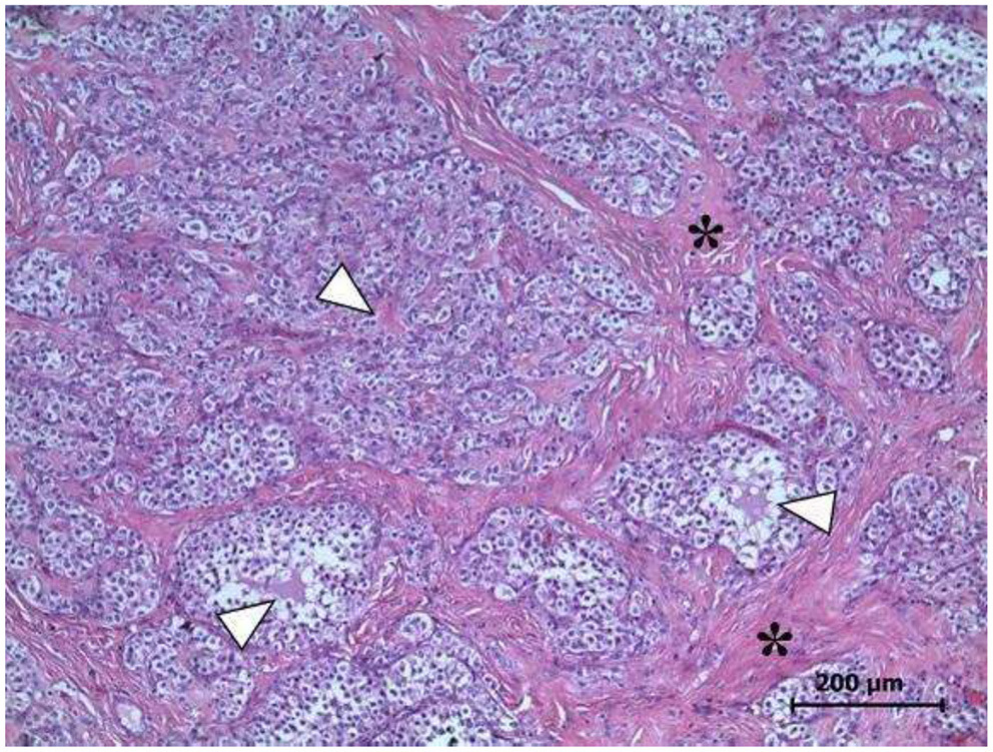
Left ovary. Microscopic evidence of GCT. The Call-Exner bodies, pathognomonic of GCT (arrowheads), and abundant stromal tissue (asterisks) are observed. Several areas exhibit the typical Sertoli pattern H&E Staining, 400X. Scale bar: 200 μm.

The histopathological exam evidenced a GCT in the left ovary, GCT in a solid tubular pattern containing Call-Exner bodies, tubular structures typical of a Sertoli pattern, surrounded by abundant stromal tissue (Figure 3). The neoplastic cells exhibited scare cytoplasm, cytoplasmic projections with rounded or oval nuclei, hyperchromatic or clear, having one or several nucleoli. Similarly, two mitotic figures per 400X field were observed.

**Figure 4:**
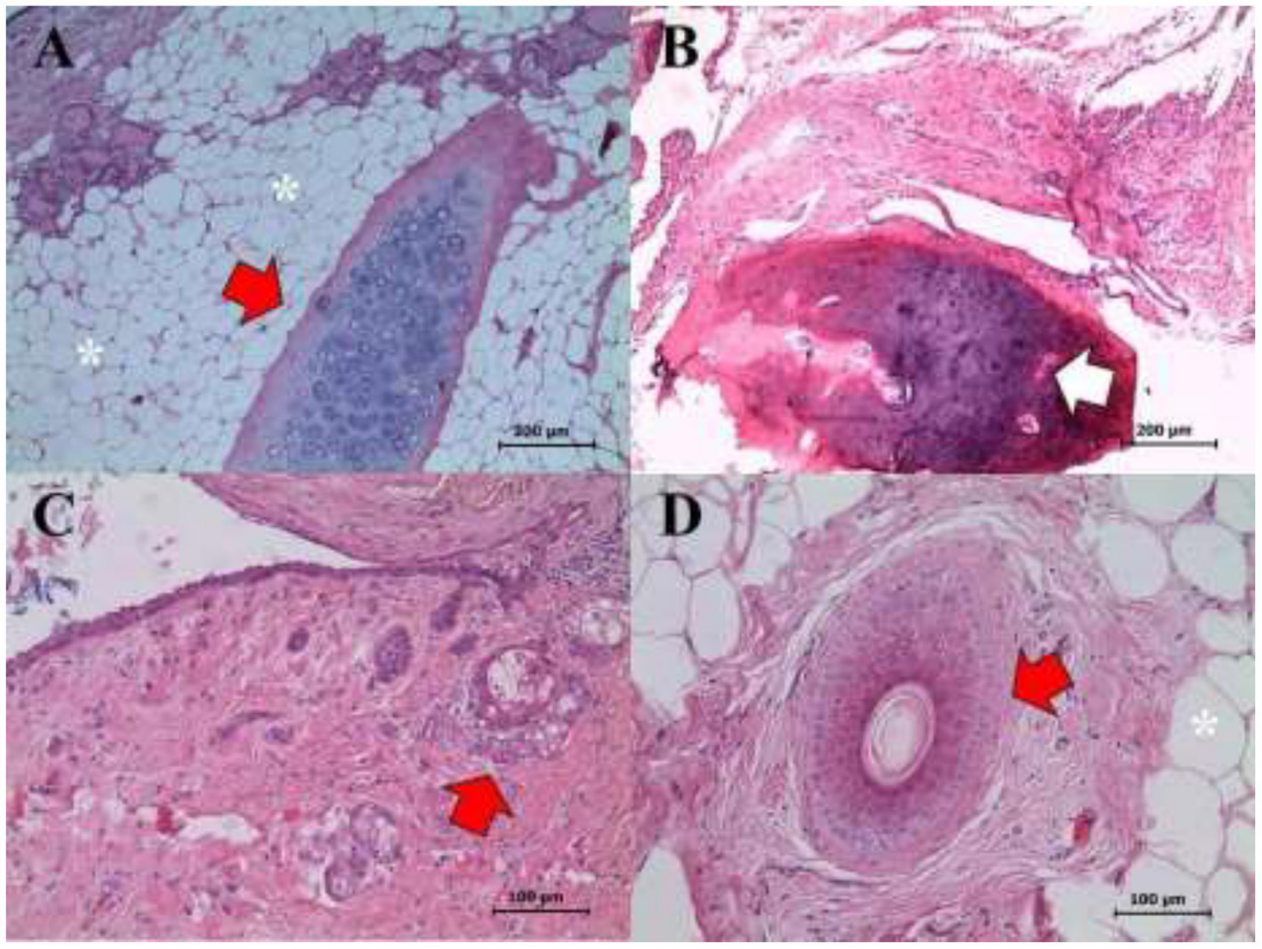
Right ovary. Mature and well-differentiated teratoma showing the following structures: **(A)** Cartilage (red arrow), glandular tissue (see eosinophilic content in the upper left corner), and adipose tissue (asterisks) (H&E staining, 400X). **(B)** Bone tissue (white arrow) (H&E staining, 200X). **(C)** Cystic hair follicle (red arrow) (H&E staining, 400X). **(D)** Cartilage (red arrow), and adipose tissue (asterisks) (H&E staining, 400X). Scale bars: 100 and 200 μm.

**Figure 5:**
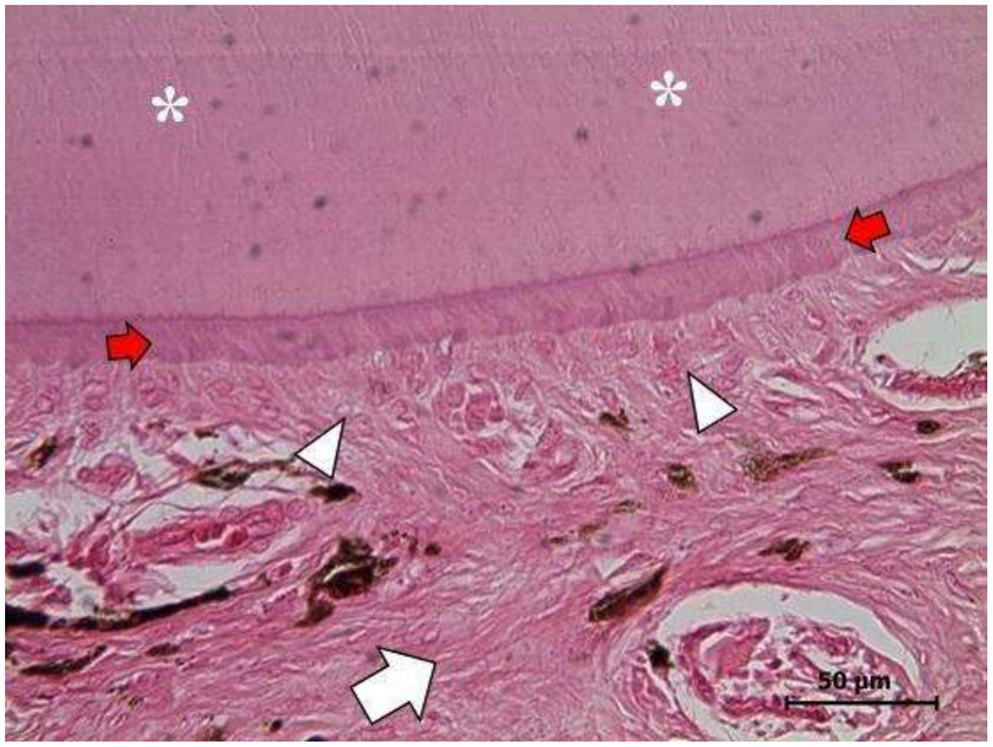
Right ovary. Mature well-differentiated teratoma exhibiting a dentine-pulp complex: dentine filaments (asterisks), pre-dentine zone (red arrows), the odontoblast layer (white arrowheads), and the pulp zone (white arrow). (H&E staining, 1,000X). Scale bars: 50 μm.

In the right ovary, a well-differentiated teratoma was found (Figures 4, 5) that included fragments of perichondrium-containing hyaline cartilage (Figure 4A), trabecular and compact bone (Figure 4B), smooth muscle, sweat glands, hair follicles, stratified cylindrical, and simple plane epithelium. Abundant cavities covered by a stratified keratinized flat epithelium, containing abundant keratin, and supported by a dense connective tissue and adipose tissue were found (Figures 4C,D). Finally, a dentin-pulp complex containing dentine, pre-dentine, odontoblasts, and pulp was found (Figure 5). Accordingly, the definite diagnosis was teratoma of the right ovary, GCT of the left ovary, and open-cervix pyometra.

## Discussion

This report present the concomitant presence of teratoma, GCT, and pyometra in a female dog referred to the University of Antioquia VTH, in agreement with previous reports (3, 15). The only remarkable clinicopathological finding at consultation was normocytic normochromic anemia and lymphopenia. Anemia could be the result of a pyometra-related sub-acute to a chronic condition, due to depressed erythropoiesis. Three weeks after surgery, the anemia was solved, as previously reported (9). Caution must be kept because some GCT are hormonally active and could induce hyperestrinism and aplastic anemia (16). In this report we could not established if there was a direct causality of the GCT and teratoma, and no reports about this relationship were found in the literature.

Granulosa cell tumors are the most frequently diagnosed ovarian tumor in bitches, representing up to 50% of tumor in female dogs, and arise from the granulosa cells in the tertiary follicles (9). In the present report, the dog had presented estrus 3 weeks before consultation consistent with the GCT deriving from the tertiary follicles. Age and reproductive status of the affected dogs appear to be related to the development of ovarian tumors (17).

In some cases, ovarian neoplasia arises at an earlier age (15). It appears that the left ovary is more frequently affected compared to the right ovary (18). Even though alpha-inhibin appears to be a useful marker for the diagnosis of GCT in dogs (13), lack of available laboratories offering this service in veterinary settings is a limiting factor for its implementation as a diagnostic tool. Recently, it was presented evidence on the usefulness of elevated anti-Mullerian hormone concentrations indicative for a GCT in bitches was presented (12). Only changes in size and macroscopic appearance of the ovaries could guide the surgeon to suspect ovarian abnormalities. However, most of these changes occur at the microscopic level, before being evident at the macroscopic level, commonly at an advanced age of the patient. In the present report, the affected dog presented visible evidence of abnormal uterine and ovarian findings at the laparotomy exam. Otherwise, in most OHE surgeries, ovaries and uteri are not thoroughly evaluated on a routine basis. A radiological exam could help the practitioner to diagnose or suspect a case of ovarian teratoma if the developed tissue harbor mineralized structures. The ultrasound exam allows the practitioner to detect some ovarian neoplasia depending on the characteristics and localization of the mass to evaluate (19). The confirmatory diagnosis of ovarian tumors is achieved only by histopathologic evaluation (9). A combination of clinical exam, ultrasonography, laparotomy, and histopathology, is the most accurate approach for diagnosing this type of tumors (16).

One of the most relevant findings in the present report is the concomitant presence of GCT and teratoma in the left and right ovaries, respectively, which could be related to the finding of open-cervix pyometra, presented by the dog at the consultation. After the report by Coggeshall et al. (15), this is the second known report on the concomitant presence of two different types of tumors in the ovaries of the same dog.

Regarding the origin of two different types of tumors in the ovaries of the same individual, there are two reports with similar findings in women (20, 21). Ovariohysterectomy is the first therapeutic decision for dogs concomitantly suffering from ovarian tumors and pyometra complex. Adjuvant treatment of ovarian tumors in the dog is not recommended, because the prognosis of ovarian tumors is the same independently of the type of histologic classification (19). Accordingly, it is always recommended to perform OHE.

## Conclusions

The frequency of ovarian neoplasia in dogs is low, in some way underdiagnosed, and represents up to 1.2% of neoplasia in this species (22). Granulosa cell tumors can cause hormonal disbalances due to the effect of the anti-Mullerian hormone in the hypophyseal-gonadotropic axis (11, 13). Treatment of ovarian neoplasia by OHE results in favorable prognosis, if there are no malignancies; malignancies do, however, occur in up to 20% of cases. Diagnosing of canine ovarian tumors is a difficult task due to discrete and non-specific clinical symptoms and the coexistence of other reproductive problems that mask the primary diagnosis, as is the case of pyometra. Also, the diagnosis of ovarian neoplasia is, in most cases, accidental during an elective or therapeutic surgical procedure. It is infrequent to find two types of ovarian tumors in the ovaries of the same dog, particularly in the absence of previous exogenous steroid hormone treatment. Care must be kept at the consultation when attending non-spayed dogs at advanced ages (more than 6 years old), which would probably be at high risk of suffering from undetected ovarian tumors, whose malignancy index increased proportionally with age and non-spaying.

## Data Availability Statement

All datasets generated for this study are included in the article.

## Ethics Statement

Ethical review and approval was not required for the animal study because this is a case report. Even though the owner signed an informed consent. Written informed consent was obtained from the owners for the participation of their animals in this study.

## Author Contributions

CO-P conceived the manuscript and performed the surgery with LH and CR-B. JM-E wrote the manuscript. All authors participated in the critical review of the manuscript.

### Conflict of Interest

The authors declare that the research was conducted in the absence of any commercial or financial relationships that could be construed as a potential conflict of interest.
